# Real-world outcomes for selumetinib in pediatric patients with neurofibromatosis type 1 and plexiform neurofibromas in Japan: A 1-year interim analysis

**DOI:** 10.1093/noajnl/vdag042

**Published:** 2026-02-16

**Authors:** Yoshihiro Nishida, Akiyo Kitajima, Tomonori Ishii

**Affiliations:** Department of Rehabilitation, Nagoya University Hospital, Nagoya, Japan; Patient Safety, Alexion Pharma GK, Tokyo, Japan; Medical Affairs, Alexion Pharma GK, Tokyo, Japan

**Keywords:** Japan, neurofibromatosis type 1, plexiform neurofibromas, postmarketing surveillance data, selumetinib

## Abstract

**Background:**

This postmarketing surveillance assessed the real-world safety and effectiveness of selumetinib in pediatric patients with neurofibromatosis type 1 (NF1) and plexiform neurofibromas (PN) in Japan.

**Methods:**

The surveillance was initiated on November 16, 2022, the launch date of selumetinib in pediatric indication in Japan. All patients treated with selumetinib were eligible for enrollment. The observation period was set to 3 years from drug initiation (data cutoff for 1-year analysis: October 9, 2024). Patient characteristics and safety and effectiveness data were captured using case report forms.

**Results:**

In total, 52 patients were included in the analysis (median [range] age, 13.0 [5–20] years; age <19 years, 49 [94.2%] patients; female, 28 [53.8%] patients; dermatological, neurological, and bone manifestations [DNB] classification stage 5, 30 [57.7%] patients; median disease duration [*N* = 49], 118.0 months). The median treatment duration was 52.1 weeks, and 46 (88.5%) patients were on selumetinib treatment at 1 year. Adverse drug reactions (ADRs) and serious ADRs were observed in 46 (88.5%) and 9 (17.3%) patients, respectively. The most common ADRs were diarrhoea (28.8%), dermatitis acneiform (26.9%), blood creatine phosphokinase increased (23.1%), and paronychia (19.2%). No fatal ADRs were reported. The median time to the first onset of any ADR was 0.492 months. Investigator’s assessment, target PN size on imaging, patient’s general self-assessment, and performance status generally indicated the effectiveness of selumetinib.

**Conclusions:**

The safety profile of selumetinib was generally consistent with the findings of the phase 2 SPRINT trial and Japanese phase 1 trial. No new safety concerns were identified.

Key Points

- This postmarketing surveillance reports real-world outcome of selumetinib in Japan.
- Selumetinib was well tolerated without new safety concerns in the interim analysis.
- The results are in line with the findings of phase 2 and 1 trials of selumetinib.

Importance of the Study

This 1-year interim analysis of an all-case postmarketing surveillance assessed the safety and effectiveness of selumetinib in 52 Japanese pediatric patients with neurofibromatosis type 1 and plexiform neurofibromas (PN) whose case report forms were fixed. The most common adverse drug reactions (ADRs) were diarrhoea, dermatitis acneiform, increased blood creatine phosphokinase, and paronychia. Most of the reported ADRs were nonserious. Our results also indicated that the timing of the first onset and frequency of occurrence may differ by ADR. Investigator’s assessment of PN symptoms, target PN size on imaging, patient’s general self-assessment, and performance status generally indicated the effectiveness of selumetinib. The safety profile of selumetinib was largely consistent with the findings of the phase 2 SPRINT and Japanese phase 1 trials. No new safety ­concerns have been identified. The results of the final 3-year analysis will provide further insights into the safety and effectiveness of selumetinib in real-world clinical practice.

Neurofibromatosis type 1 (NF1) is an autosomal dominant, multisystem disorder.^1^ The disease is caused by loss-of-function mutations in the *NF1* gene that lead to unregulated RAS pathway activation and aberrant cell proliferation.^1^ NF1 is one of the most common genetic disorders worldwide,^1^ with a penetrance close to 100%^1^ and an estimated prevalence of approximately 1 in 3000 persons.^2^^,^^3^ Approximately half of patients with NF1 have plexiform neurofibromas (PN).^1^ Symptoms of NF1 and PN may occur during infancy or early childhood,^1^ posing a significant clinical and humanistic burden on patients and their caregivers.^4^ Patients commonly present with NF1-related findings, such as café-au-lait macules, and occasionally have PN-associated findings, such as cosmetic deformation, pain, and impaired function.^5^ Surgery has traditionally been the standard treatment for symptomatic PN. In some cases, debulking surgeries are performed for symptom management but are often associated with undesirable sequelae.^4^

Selumetinib, an orally administered, selective, mitogen-activated protein kinase (MEK) 1/2 inhibitor, is the first medical treatment approved for pediatric patients with NF1 and symptomatic inoperable PN.^6-8^ Selumetinib is available for treatment in the United States,^6^ the European Union,^7^ Japan,^8^ and many other countries and recommended for use in various treatment guidelines.^3^^,^^9-11^ The efficacy and safety of selumetinib in children with NF1 and symptomatic, inoperable PN have been demonstrated in the open-label, phase 2 SPRINT trial (ClinicalTrials.gov identifier, NCT01362803).^12^ In addition, selumetinib was well tolerated in Japanese (ClinicalTrials.gov identifier, NCT04495127)^13^ and Chinese (ClinicalTrials.gov identifier, NCT04590235) phase 1 clinical trials.^14^ Selumetinib demonstrated efficacy without new safety concerns in adults with NF1 and symptomatic, inoperable PN in the phase 3 KOMET trial (ClinicalTrials.gov identifier, NCT04924608).^15^ The drug also showed efficacy and a manageable safety profile in pediatric and adult patients in South Korea^16^ and children with NF1 and inoperable PN without clinically significant morbidity,^17^ both in phase 2 trial settings.

The safety and efficacy profile of selumetinib has been described in a quantitative review that pooled data from 134 adult and pediatric patients in 6 uncontrolled trials.^18^ The most common adverse events (AEs) reported therein were grade 1 or 2 gastrointestinal symptoms (65%), asymptomatic creatine phosphokinase increase (31%), acneiform rash (17%), and paronychia (6%).^18^ Although AEs potentially associated with selumetinib are generally manageable, the risk-benefit balance should be carefully considered for clinical use of the drug, and further investigation on its safety profile is warranted.^19^ Small-scale studies have indicated the effectiveness of selumetinib in real-world clinical practice,^20^^,^^21^ but evidence for its long-term use in a large pediatric patient population is insufficient.

This postmarketing surveillance (PMS) aimed to assess the safety and effectiveness of selumetinib in a larger pediatric population receiving treatment in Japanese real-world clinical practice and capture the clinical characteristics of patients treated with selumetinib in Japan. Herein, we present the results from a 1-year interim analysis.

## Patients and Methods

### Study Design and Eligibility Criteria

This PMS was designed as a noninterventional, single-cohort, observational, all-case surveillance with an observation period of 3 years from drug initiation. The surveillance was initiated on November 16, 2022, the launch date of selumetinib for pediatric indication in Japan. The surveillance period and patient enrollment period were initially set to 8 years and 4 years, respectively, from the drug launch date. Patient enrollment was completed on December 11, 2024. Data cutoff for the 1-year interim analysis was set to October 9, 2024. In principle, all patients who were treated with selumetinib were eligible to participate in the surveillance regardless of age per the regulatory approval condition of the drug in Japan. No exclusion criteria were set.

### Treatment

Patients were treated with selumetinib in real-world clinical practice. The dose for selumetinib in Japan described in the drug package insert is twice daily, 25 mg/m^2^ body surface, administered in a fasting state.^8^ The dose can be adjusted based on the patient’s condition, with a maximum dose of 50 mg per dosing.^8^ In this surveillance, selumetinib was administered based on the investigator’s discretion in consultation with patients and their families. Dose changes, interruptions, resumptions, or discontinuations were also based on the investigator’s discretion.

### Assessments and Outcomes

Patient data were captured at the initiation of selumetinib treatment and after 1 year of selumetinib treatment using case report forms (CRFs). At the initiation of selumetinib treatment, patient demographics and clinical characteristics were recorded. The Japanese dermatological, neurological, and bone manifestations (DNB) classification,^22^ created by the Neurocutaneous Syndrome Research Group in Japan, was used to assess disease severity. The DNB classification consists of the dermatological (D1 [pigmented macules and a few neurofibromas]–D4 [severe plexiform neurofibromas or malignant peripheral nerve sheath tumor]), neurological (N0 [no neurological symptoms]–N2 [severe or progressive neurological symptoms]), and bone (B0 [no bone lesion]–B2 [severe bone lesion that needs surgical treatment, severe bone deformity in the extremities, or defect of the skull or facial bone]) manifestations. Patients are classified into stages 1 (no problems in daily life and social activity) to 5 (severe problems in daily life due to physical abnormality) based on the combination of the 3 manifestations.^22^ For example, a combination of D1, N0, and B0 corresponds to DNB classification stage 1, whereas a combination of D3, N0, and B0 corresponds to DNB classification stage 3. A patient is classified as DNB classification stage 5 if the manifestations include D4, N2, or B2.^22^ Patients’ performance status at baseline was evaluated using the Lansky play-performance status (100 [fully active, normal] to 10 [does not play nor get out of bed]) for those aged ≤16 years.^23^ The Karnofsky performance status (100 [normal, no complaints, no evidence of disease] to 10 [moribund, fatal processes progressing rapidly]) was used for those aged ≥17 years. The type (nodal or diffuse) and status (progressive or nonprogressive) of PN were recorded based on the investigator’s assessment. In addition, information regarding selumetinib dose and concomitant drugs or therapies was captured.

The incidence of adverse drug reactions (ADRs) and serious ADRs was assessed as the safety outcome. ADRs were defined as AEs for which a causal relationship with selumetinib could not be excluded by the investigator or study sponsor. Seriousness of ADRs was assessed based on the post-approval safety data management guidelines (E2D guidelines) set forth by the International Council for Harmonisation of Technical Requirements for Pharmaceuticals for Human Use (ICH).^24^^,^^25^ In brief, ADRs that resulted in death, were life threatening, resulted in persistent or significant disability or incapacity, required hospitalization or prolongation of existing hospitalization, or were deemed as other medically significant conditions, congenital abnormality, or birth defect were recorded as serious ADRs. ADRs and serious ADRs were coded using the Medical Dictionary for Regulatory Activities (MedDRA) Japanese version 27.0.

The outcome of the first ADR onset was recorded as recovered/resolved, recovering/resolving, not recovered/not resolved, recovered/resolved with sequelae, fatal, or unknown based on the message specification of electronic transmission of individual case safety reporting (E2B M2 specification document) set forth by the ICH.^24^^,^^26^ In addition, the time to the first onset of any ADR was analyzed. The same analyses on the outcomes and time to the first onset were performed for dermatitis acneiform, paronychia, and gastrointestinal symptoms (nausea, vomiting, or diarrhoea) as they were the AEs commonly observed in past clinical trials of selumetinib.^12^^,^^13^ To explore the baseline characteristics that may affect the onset of ADRs, patients were stratified into subgroups based on baseline characteristics, and the incidence of ADRs was compared across the subgroups.

Effectiveness of selumetinib at 1 year was assessed using the following outcomes. The overall evaluation of PN was performed by the investigator based on the changes in the signs, symptoms, and complications of the disease, and the result was recorded as improved, unchanged, or worsened. The target PN size was evaluated based on magnetic resonance imaging (MRI), computed tomography (CT), or echography data and was recorded as decreased, unchanged, or increased by the investigator. Patients were asked to provide general self-assessment on their symptoms (improved, unchanged, or worsened), and the results were captured in the CRF by the investigator. Performance status (Lansky play-performance status for those aged ≤16 years, Karnofsky performance status for those aged ≥17 years) was assessed by the investigator and recorded in the CRF.

### Statistical Analyses

A sample size of 300 patients for the safety analysis was set based on the feasibility of the surveillance. With this sample size, serious AEs with an estimated incidence rate of 4.2% could be detected with a one-sided significance level of 2.5% and a power of 80%.

Among the patients enrolled in the PMS, those who had completed CRFs and provided written informed consent for data publication were included in the analysis. Patients with inadequate research contracts at the institutions, inadequacy in patient enrollment procedures, or incomplete information on CRFs were excluded from the safety analysis. Patients without a selumetinib dose, those who had previously used selumetinib through private drug import, or those with no evaluable data for safety were also excluded from the safety analysis. Among patients included in the safety analysis, those who had no evaluable data for effectiveness or who had been treated with selumetinib in previous clinical trials were excluded from the effectiveness analysis.

Continuous variables were summarized as the median and range, and categorical variables were summarized as the number and proportion of patients. Time to the first ADR onset was analyzed using the Kaplan–Meier method. In the subgroup analysis, the incidence of ADRs and the 95% confidence intervals (CIs) were calculated for each subgroup. Missing values were not imputed during the analysis. All analyses were performed using SAS Version 9.4 (SAS Institute, Inc., Cary, NC, USA).

## Results

### Patient Disposition

**Figure 1.**
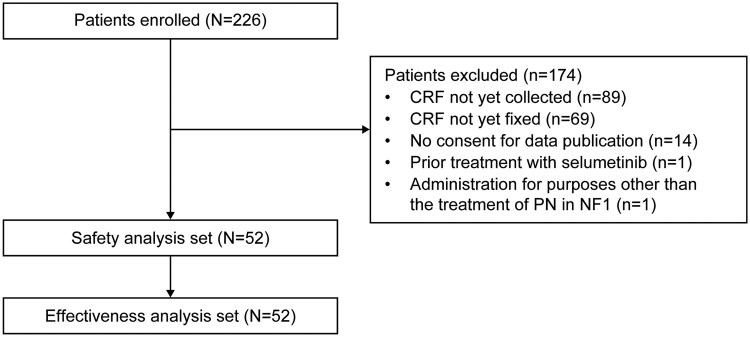
Patient disposition. Abbreviations: CRF, case report form; NF1, neurofibromatosis type 1; PN, plexiform neurofibroma.

A total of 226 patients were enrolled in this surveillance across Japan, of whom 52 were included in the safety and effectiveness analyses (Figure 1). The most common reasons for exclusion were CRFs not yet collected (89 patients) and CRFs not yet fixed (69 patients) before the data cutoff date. Fourteen patients did not provide consent for data publication and were thus excluded from the analysis (Figure 1).

### Baseline Characteristics

The median (range) patient age was 13.0 (5–20) years, and 49 (94.2%) of 52 patients were aged <19 years (Table 1). In total, 28 (53.8%) were female patients, and 30 (57.7%) patients were classified into DNB classification stage 5. Most patients with evaluable data recorded 90 to 100 on the Lansky play-performance status and 80 to 90 on the Karnofsky performance status. A total of 45 (86.5%) of the 52 patients had diffuse PN and 38 (73.1%) had progressive PN. The median disease duration among the 49 evaluable patients was 118.0 months. Target PN lesions (multiple lesions may have been recorded in a single patient) were most commonly observed in the head and neck (30 [57.7%] patients), followed by the trunk (20 [38.5%] patients) and limbs (13 [25.0%] patients). NF1 lesions other than PN were observed in 47 (90.4%) patients (Table 1). Such lesions were mostly observed in the skin (38 patients), bone (17 patients), and ocular regions (10 patients).

**Table 1.** Baseline characteristics (safety analysis set).

Variable	Safety analysis set (*N* = 52)
Age (years), median (range)	13.0 (5–20)
<19 years, *n* (%)	49 (94.2)
≥19 years, *n* (%)	3 (5.8)
Female sex, *n* (%)	28 (53.8)
Patients with medical history, *n* (%)	6 (11.5)
Patients with complications, *n* (%)	25 (48.1)
Lansky play-performance status (patients aged ≤16 years), *n* (%)	*N* = 38
≤50	0 (0.0)
60	1 (2.6)
70	2 (5.3)
80	5 (13.2)
90	9 (23.7)
100	21 (55.3)
Karnofsky performance status (patients aged ≥17 years), *n* (%)	*N* = 13
≤50	0 (0.0)
60	1 (7.7)
70	1 (7.7)
80	2 (15.4)
90	8 (61.5)
100	1 (7.7)
DNB classification, *n* (%)	
Stage 1	3 (5.8)
Stage 2	16 (30.8)
Stage 3	2 (3.8)
Stage 4	1 (1.9)
Stage 5	30 (57.7)
Type of PN, *n* (%)^a^	
Nodal (neurofibroma of nerves)	13 (25.0)
Diffuse (diffuse neurofibroma)	45 (86.5)
Status of PN, *n* (%)	
Progressive	38 (73.1)
Nonprogressive	14 (26.9)
History of surgical resection, *n* (%)	17 (32.7)
Disease duration (months; *N* = 49), median (range)	118.0 (12–227)
Target PN lesion, *n* (%)^a^	
Head and neck	30 (57.7)
Trunk	20 (38.5)
Limbs	13 (25.0)
Other	1 (1.9)
Patients with NF1 lesions other than PN, *n* (%)	47 (90.4)
Patients with prior treatment for PN, *n* (%)	13 (25.0)
Patients with concomitant treatment for NF1, *n* (%)	5 (9.6)

Abbreviations: DNB, dermatological, neurological and bone manifestations; NF1, neurofibromatosis type 1; PN, plexiform neurofibroma.

a The total number of patients exceeded 52 because some patients were categorized into more than 1 category.

### Exposure to Selumetinib

The median (range) treatment duration was 52.1 (21.0–65.1) weeks, and the median (range) total cumulative dose was 18120.0 (620–32850) mg. At 1 year, 46 (88.5%) of the 52 patients were on continued treatment with selumetinib. Six patients discontinued treatment; the reasons for discontinuation (multiple reasons may have been recorded in a single patient) were AEs in 4 patients, patient request in 2 patients, and other reasons in 1 patient.

### Safety

In total, 48 (92.3%) and 11 (21.2%) of the 52 patients had AEs and serious AEs, respectively, and ADRs were observed in 46 (88.5%) patients (Table 2). The most common ADR was diarrhoea (15 [28.8%] patients), followed by dermatitis acneiform (14 [26.9%] patients), blood creatine phosphokinase increased (12 [23.1%] patients), and paronychia (10 [19.2%] patients). Sixteen (30.8%) patients had ADRs leading to treatment discontinuation, including dose interruption and resumption (Table 2). The outcomes of the first ADR onset in the 46 patients with ADRs were recovered/resolved in 17 (37.0%), recovering/resolving in 13 (28.3%), and not recovered/not resolved in 16 (34.8%) patients. Five (50.0%) of the 10 patients with paronychia recorded not recovered/not resolved as the outcome of the ADR, while 9 (47.4%) of 19 patients with gastrointestinal symptoms recovered/resolved without sequelae. No fatal ADRs were reported (Supplementary Table S1).

**Table 2.** Incidence of AEs and ADRs (safety analysis set).

Variable	Safety analysis set (*N* = 52)
Patients with any AE, *n* (%)	48 (92.3)
Patients with any ADR, *n* (%)	46 (88.5)
Patients with any SAE, *n* (%)	11 (21.2)
Patients with any serious ADR, *n* (%)	9 (17.3)
Patients with any ADR leading to drug discontinuation^a^, *n* (%)	16 (30.8)
ADRs observed in ≥5% of the patients, *n* (%)	
Gastrointestinal disorders	
Diarrhoea	15 (28.8)
Stomatitis	7 (13.5)
Abdominal pain	6 (11.5)
Nausea	6 (11.5)
Vomiting	3 (5.8)
General disorders and administration site conditions	
Malaise	4 (7.7)
Infections and infestations	
Paronychia	10 (19.2)
Investigations	
Blood creatine phosphokinase increased	12 (23.1)
Skin and subcutaneous tissue disorders	
Dermatitis acneiform	14 (26.9)
Dermatitis	7 (13.5)
Alopecia	6 (11.5)
Eczema	4 (7.7)
Acne	3 (5.8)
Rash	3 (5.8)

Abbreviations: ADR, adverse drug reaction; AE, adverse event; SAE, serious adverse event.

a Including dose interruption and resumption.

Nine (17.3%) patients experienced serious ADRs. The most common serious ADR was blood creatine phosphokinase increased, reported in 3 (5.8%) patients, followed by paronychia in 2 (3.8%) patients. Other serious ADRs were serous retinal detachment, uveitis, diarrhoea, inguinal hernia, nausea, malaise, pain, hepatic function abnormal, moyamoya disease, pneumothorax, and dermatitis acneiform, reported in 1 (1.9%) patient each. The outcomes of all the serious ADRs were recorded as recovered/resolved without sequelae or recovering/resolving (Supplementary Table S2).

**Figure 2.**
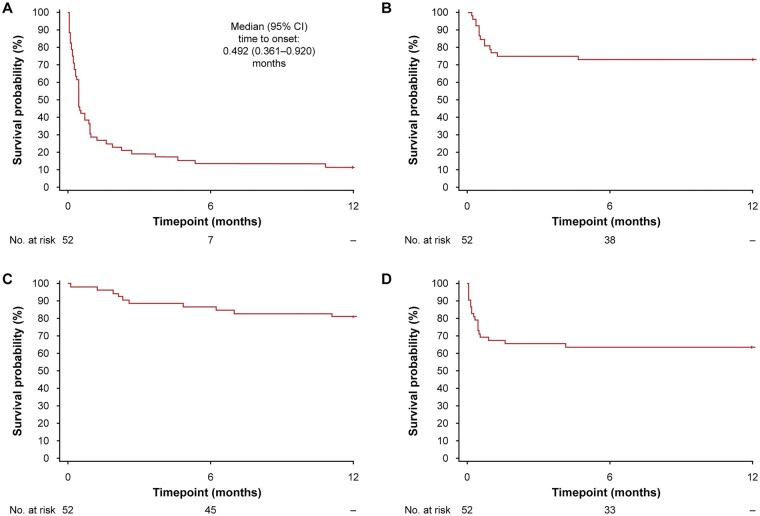
Time to the first onset of (A) any ADR, (B) dermatitis acneiform, (C) paronychia, and (D) gastrointestinal symptoms (nausea, vomiting, or diarrhoea) (safety analysis set). Abbreviations: ADR, adverse drug reaction; CI, confidence interval.

Kaplan–Meier analyses of the time to the first ADR onset indicated that most patients experienced the first onset of any ADR, dermatitis acneiform, or gastrointestinal symptoms early after the initiation of selumetinib treatment (Figure 2A–2D). The median time to the first onset was 0.492 months (95% CI, 0.361–0.920) for any ADR (Figure 2A).

In the subgroup analysis for factors that may affect the onset of ADRs, the incidence of ADRs was largely similar across subgroups (Supplementary Table S3). The results indicated that there were no patient baseline characteristics that may have affected the onset of ADRs.

### Effectiveness

**Figure 3.**
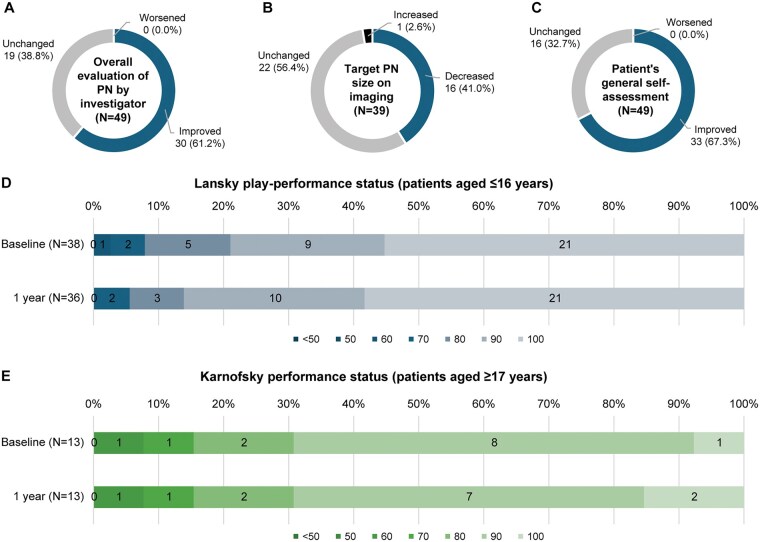
Effectiveness assessments using (A) overall evaluation of PN by the investigator, (B) target PN size on imaging, (C) patient’s general self-assessment (effectiveness analysis set), (D) Lansky play-performance status, and (E) Karnofsky performance status (safety analysis set). Abbreviation: PN, plexiform neurofibroma.

At 1 year, the effectiveness assessments showed a trend toward improvement in the overall evaluation of PN performed by the investigator (Figure 3A), decrease in the target PN size on imaging (Figure 3B), and patient’s general self-assessment (Figure 3C) with selumetinib treatment. The results also indicated sustained Lansky play-performance status (Figure 3D; patients aged ≤16 years) or Karnofsky performance status (Figure 3E; patients aged ≥17 years) with selumetinib treatment.

## Discussion

Selumetinib was approved in Japan in 2022 as the first and only medical treatment for pediatric patients (aged 3–19 years) with NF1 and symptomatic, inoperable PN.^8^ This PMS aimed to assess the safety and effectiveness of selumetinib in patients with NF1 and PN in Japanese real-world clinical practice. Overall, the safety findings derived from this surveillance were consistent with those of previous phase 2 or Japanese phase 1 trials of selumetinib.^12^^,^^13^^,^^17^

The patient age ranged from 5 to 20 years (median 13.0 years), which is slightly higher than the age of the phase 2 SPRINT trial participants (range 3.5–17.4 years, median 10.2 years).^12^ This surveillance included 3 patients aged ≥19 years, owing to their transition from previous clinical trials of selumetinib and the adoption of an all-case surveillance design per the regulatory approval condition of selumetinib in Japan. Most patients recorded 70 to 100 on the Lansky play-performance status or Karnofsky performance status in the current surveillance, which is similar to the data derived from the phase 2 trial in South Korea.^16^ Target PN lesions were commonly observed in the head, neck, and trunk, in line with a 2007–2018 Japanese retrospective survey of 40 patients with NF1 and diffuse PN.^27^ More than half (57.7%) of the patients in this PMS were classified into DNB stage 5. This prevalence is higher than that reported in a Japanese single-center retrospective study (33.9%),^22^ indicating an advanced disease stage in the patients involved in the current surveillance.

The ADRs most commonly observed in this surveillance were diarrhoea, dermatitis acneiform, blood creatine phosphokinase increased, and paronychia. These findings were largely similar with the AEs reported in previous phase 2 and Japanese phase 1 trials of selumetinib.^12^^,^^13^^,^^17^ Furthermore, the most common ADRs are consistent with those from a retrospective pharmacovigilance study of the Food and Drug Administration Adverse Event Reporting System (FAERS), which indicates the importance of addressing AEs associated with selumetinib early, particularly within the initial month of treatment.^28^ In contrast, 5-year data from the phase 1 and phase 2 clinical trials of selumetinib in children suggest that patients may develop drug-related AEs, such as asymptomatic decrease in left ventricular ejection function, several years after treatment.^29^ In the current surveillance, analyses of the time to the first ADR onset indicated that most patients experienced the first onset of any ADR, dermatitis acneiform, or gastrointestinal symptoms early after the initiation of selumetinib treatment. According to a quantitative review of selumetinib, 17% and 10.5% of clinical trial participants required dose reduction and treatment discontinuation, respectively, because of AEs.^18^ Data on the timing of ADR onset may help clinical decision-making in terms of treatment choice, dose reduction, or treatment termination. The final 3-year results of this PMS are awaited.

This PMS observed 1 patient who had serous retinal detachment and uveitis as serious ADRs, which were both recorded as recovering/resolving with selumetinib dose interruption. Ocular effects are commonly observed in patients treated with MEK inhibitors, and most of them are asymptomatic or benign; however, some patients may experience moderate or severe toxicities associated with visual symptoms.^30^ In the phase 2 SPRINT trial, none of the participants had serous retinal detachment or other ocular events that were vision-threatening.^12^ In contrast, central serous retinopathy has been reported in one patient each in a phase 1 trial of selumetinib^29^ and a case series conducted in Japan.^20^ Eye disorders are listed as an important identified risk in the Japanese Risk Management Plan for selumetinib,^31^ and further investigation is needed in the final 3-year analysis to add additional insights into potential ocular effects.

Overall, the results of this PMS indicated effectiveness of 1-year selumetinib treatment. However, the effectiveness was evaluated primarily based on the assessments by the investigators and patients. Uniform international criteria, central assessment, or validated patient-reported outcome (PRO) instruments were not applied. Furthermore, a direct comparison of effectiveness with other clinical trial data is not feasible because of the variability in the scales used.

This PMS is one of the first large-scale research to assess the real-world safety of selumetinib in pediatric patients with NF1 and symptomatic, inoperable PN in Japan. Baseline characteristics derived from it can be generalizable and serve as the nationwide epidemiology data in this country. However, this surveillance has several limitations, including the use of single-arm design without comparators. As this surveillance aimed to collect safety and effectiveness data in daily clinical practice, the study sponsor was not involved in the assessments or data collection. The interpretation of the effectiveness data was limited because of the use of subjective assessments by investigators and patients, without objective measures, a central assessment, or validated PRO instruments. As this surveillance is still underway, a time lag between patient enrollment and CRF collection is inevitable, leading to a relatively small proportion of enrolled patients being eligible for the 1-year interim analysis. Moreover, patients’ quality of life, pain intensity, or neurocognitive function was not assessed. Lastly, this is an interim analysis with a follow-up period of 1 year. The findings derived from the current analysis may change after the final 3-year analysis is completed.

In conclusion, the safety profile of selumetinib was generally consistent with the findings of the phase 2 SPRINT trial and Japanese phase 1 trial of selumetinib. To date, no new safety concerns have been identified. Our results also showed preliminary effectiveness of 1-year selumetinib treatment in this 3-year surveillance. The safety and effectiveness of selumetinib continue to be assessed in patients with NF1 and PN in Japan.

## Supplementary Material

vdag042_Supplementary_Data

## Data Availability

Alexion, AstraZeneca Rare Disease will consider requests for disclosure of clinical study participant-level data provided that participant privacy is assured through methods like data de-identification, pseudonymization, or anonymization (as required by applicable law), and if such disclosure was included in the relevant study informed consent form or similar documentation. Qualified academic investigators may request participant-level clinical data and supporting documents (statistical analysis plan and protocol) pertaining to Alexion-sponsored studies. Further details regarding data availability and instructions for requesting information are available in the Alexion Clinical Trials Disclosure and Transparency Policy at https://www.alexionclinicaltrialtransparency.com/data-requests/.
