# Correction to: Real-world outcomes for selumetinib in pediatric patients with neurofibromatosis type 1 and plexiform neurofibromas in Japan: A 1-year interim analysis

**DOI:** 10.1093/noajnl/vdag214

**Published:** 2026-08-27

This is a correction to: Yoshihiro Nishida, Akiyo Kitajima, Tomonori Ishii, Real-world outcomes for selumetinib in pediatric patients with neurofibromatosis type 1 and plexiform neurofibromas in Japan: A 1-year interim analysis, *Neuro-Oncology Advances*, Volume 8, Issue 1, January-December 2026, vdag042, https://doi.org/10.1093/noajnl/vdag042

In the originally published version of this manuscript, a zip file containing the supplementary material erroneously included differing duplicate versions.

This error has been corrected and only the correct supplementary material remains.

