# Supplementary material for: Real-world outcomes for selumetinib in pediatric patients with neurofibromatosis type 1 and plexiform neurofibromas in Japan: A 1-year interim analysis
Source: Neurooncol Adv. 2026 Feb 16;8(1):vdag042. doi: 10.1093/noajnl/vdag042 (PMC12990300; doi:10.1093/noajnl/vdag042)
Supplement: vdag042_Supplementary_Data [file vdag042_supplementary_data.docx]

**Supplementary Materials**

**Supplementary Table 1.** Outcomes of the first ADR onset (safety analysis set)

| **Type of the first ADR** | **Number of patients with ADRs, n** | **Outcome of the first ADR, n (%)** | | | | | |
| --- | --- | --- | --- | --- | --- | --- | --- |
|  |  | **Recovered/**  **resolved** | **Recovering/**  **resolving** | **Not recovered/**  **not resolved** | **Recovered/**  **resolved with sequelae** | **Fatal** | **Unknown** |
| Any ADR | 46 | 17 (37.0) | 13 (28.3) | 16 (34.8) | 0 (0.0) | 0 (0.0) | 0 (0.0) |
| Dermatitis acneiform | 14 | 4 (28.6) | 5 (35.7) | 5 (35.7) | 0 (0.0) | 0 (0.0) | 0 (0.0) |
| Paronychia | 10 | 3 (30.0) | 2 (20.0) | 5 (50.0) | 0 (0.0) | 0 (0.0) | 0 (0.0) |
| Gastrointestinal symptoms | 19 | 9 (47.4) | 6 (31.6) | 2 (10.5) | 0 (0.0) | 0 (0.0) | 2 (10.5) |
| Nausea | 6 | 3 (50.0) | 2 (33.3) | 1 (16.7) | 0 (0.0) | 0 (0.0) | 0 (0.0) |
| Vomiting | 3 | 2 (66.7) | 1 (33.3) | 0 (0.0) | 0 (0.0) | 0 (0.0) | 0 (0.0) |
| Diarrhoea | 15 | 6 (40.0) | 5 (33.3) | 2 (13.3) | 0 (0.0) | 0 (0.0) | 2 (13.3) |

Abbreviations: ADR, adverse drug reaction.

**Supplementary Table 2.** Summary of serious ADRs (safety analysis set)

| **No.** | **Age, sex** | **Serious ADR** | **Day of onset^a^** | **Selumetinib dosing** | **Other action taken** | **Outcome** |
| --- | --- | --- | --- | --- | --- | --- |
| 1 | 20 years, female | Paronychia | Day 4 | Discontinued | Drug prescription^b^ | Recovered/resolved |
| 2 | 18 years, female | Serous retinal detachment | Day 169 | Dose interrupted, resumed | None | Recovering/resolving |
|  |  | Uveitis | Day 169 | Dose interrupted, resumed | None | Recovering/resolving |
| 3 | 14 years, female | Hepatic function abnormal | Day 27 | Dose interrupted, resumed | None | Recovering/resolving |
| 4 | 17 years, female | Moyamoya disease | Day 3 | Discontinued | Cerebral neovascularization surgery | Recovered/resolved |
| 5 | 16 years, female | Dermatitis acneiform | Day 30 | No change | Drug prescription^c^ | Recovering/resolving |
|  |  | Paronychia | Day 213 | No change | Drug prescription^d^ | Recovering/resolving |
|  |  | Blood creatine phosphokinase increased | Day 269 | Dose interrupted, resumed | None | Recovered/resolved |
| 6 | 8 years, female | Blood creatine phosphokinase increased | Day 79 | Dose interrupted, resumed | None | Recovered/resolved |
| 7 | 8 years, male | Blood creatine phosphokinase increased | Day 261 | Dose interrupted, resumed | None | Recovered/resolved |
| 8 | 19 years, male | Inguinal hernia | Day 309 | No change | Drug prescription^e^, surgery | Recovered/resolved |
| 9 | 16 years, male | Diarrhoea | Day 2 | Dose interrupted, resumed | None | Recovered/resolved |
|  |  | Malaise | Day 2 | Dose interrupted, resumed | Bed rest | Recovered/resolved |
|  |  | Nausea | Day 2 | Dose interrupted, resumed | None | Recovered/resolved |
|  |  | Pain | Day 2 | Dose interrupted, resumed | Bed rest | Recovered/resolved |
|  |  | Pneumothorax | Day 80 | No change | Unknown^f^ | Recovered/resolved |

Abbreviations: ADR, adverse drug reaction.

^a^Day 1 is the day of selumetinib initiation.

^b^Gentamicin sulfate ointment, clobetasol propionate ointment, and minocycline capsule.

^c^Benzoyl peroxide gel.

^d^Betamethasone butyrate propionate ointment.

^e^Cefmetazole sodium.

^f^Treated at other hospitals.

**Supplementary Table 3.** Incidence of ADRs stratified by baseline characteristics (safety analysis set)

| **Category** | **Patients categorized, n (%) (N=52)** | **Patients with ADR, n (%)** | **95% CI of incidence** |
| --- | --- | --- | --- |
| Age | | | |
| <19 years | 49 (94.2) | 43 (87.8) | [75.23–95.37] |
| ≥19 years | 3 (5.8) | 3 (100.0) | [29.24–100.00] |
| Sex | | | |
| Male | 24 (46.2) | 22 (91.7) | [73.00–98.97] |
| Female | 28 (53.8) | 24 (85.7) | [67.33–95.97] |
| Lansky play-performance status (patients aged ≤16 years, N=38) | | | |
| ≤50 | 0 (0.0) | - | - |
| 60 | 1 (2.6) | 1 (100.0) | [2.50–100.00] |
| 70 | 2 (5.3) | 2 (100.0) | [15.81–100.00] |
| 80 | 5 (13.2) | 4 (80.0) | [28.36–99.49] |
| 90 | 9 (23.7) | 8 (88.9) | [51.75–99.72] |
| 100 | 21 (55.3) | 17 (81.0) | [58.09–94.55] |
| Karnofsky performance status (patients aged ≥17 years, N=13) | | | |
| ≤50 | 0 (0.0) | - | - |
| 60 | 1 (7.7) | 1 (100.0) | [2.50–100.00] |
| 70 | 1 (7.7) | 1 (100.0) | [2.50–100.00] |
| 80 | 2 (15.4) | 2 (100.0) | [15.81–100.00] |
| 90 | 8 (61.5) | 8 (100.0) | [63.06–100.00] |
| 100 | 1 (7.7) | 1 (100.0) | [2.50–100.00] |
| Medical history | | | |
| Yes | 6 (11.5) | 6 (100.0) | [54.07–100.00] |
| No | 46 (88.5) | 40 (87.0) | [73.74–95.06] |
| Complications | | | |
| Yes | 25 (48.1) | 22 (88.0) | [68.78–97.45] |
| No | 27 (51.9) | 24 (88.9) | [70.84–97.65] |
| DNB classification, dermatological manifestations | | | |
| D1 | 16 (30.8) | 16 (100.0) | [79.41–100.00] |
| D2 | 11 (21.2) | 10 (90.9) | [58.72–99.77] |
| D3 | 4 (7.7) | 2 (50.0) | [6.76–93.24] |
| D4 | 21 (40.4) | 18 (85.7) | [63.66–96.95] |
| DNB classification, neurological manifestations | | | |
| N0 | 19 (36.5) | 17 (89.5) | [66.86–98.70] |
| N1 | 26 (50.0) | 24 (92.3) | [74.87–99.05] |
| N2 | 7 (13.5) | 5 (71.4) | [29.04–96.33] |
| DNB classification, bone manifestations | | | |
| B0 | 28 (53.8) | 23 (82.1) | [63.11–93.94] |
| B1 | 15 (28.8) | 14 (93.3) | [68.05–99.83] |
| B2 | 9 (17.3) | 9 (100.0) | [66.37–100.00] |
| DNB classification | | | |
| Stage 1 | 3 (5.8) | 3 (100.0) | [29.24–100.00] |
| Stage 2 | 16 (30.8) | 15 (93.8) | [69.77–99.84] |
| Stage 3 | 2 (3.8) | 1 (50.0) | [1.26–98.74] |
| Stage 4 | 1 (1.9) | 1 (100.0) | [2.50–100.00] |
| Stage 5 | 30 (57.7) | 26 (86.7) | [69.28–96.24] |
| Type of PN | | | |
| Nodal (neurofibroma of nerves) | 13 (25.0) | 12 (92.3) | [63.97–99.81] |
| Diffuse (diffuse neurofibroma) | 45 (86.5) | 40 (88.9) | [75.95–96.29] |
| Status of PN | | | |
| Progressive | 38 (73.1) | 33 (86.8) | [71.91–95.59] |
| Nonprogressive | 14 (26.9) | 13 (92.9) | [66.13–99.82] |
| History of surgical resection | | | |
| Yes | 17 (32.7) | 15 (88.2) | [63.56–98.54] |
| No | 35 (67.3) | 31 (88.6) | [73.26–96.80] |
| Target PN lesion | | | |
| Head and neck | 30 (57.7) | 24 (80.0) | [61.43–92.29] |
| Trunk | 20 (38.5) | 19 (95.0) | [75.13–99.87] |
| Limbs | 13 (25.0) | 12 (92.3) | [63.97–99.81] |
| Other | 1 (1.9) | 1 (100.0) | [2.50–100.00] |
| NF1 lesions other than PN | | | |
| Yes | 47 (90.4) | 43 (91.5) | [79.62–97.63] |
| No | 5 (9.6) | 3 (60.0) | [14.66–94.73] |
| Prior treatment for PN | | | |
| Yes | 13 (25.0) | 12 (92.3) | [63.97–99.81] |
| No | 38 (73.1) | 33 (86.8) | [71.91–95.59] |
| Unknown | 1 (1.9) | 1 (100.0) | [2.50–100.00] |

Abbreviations: ADR, adverse drug reaction; CI, confidence interval; DNB, dermatological, neurological and bone manifestations; NF1, neurofibromatosis type 1; PN, plexiform neurofibroma.
